# Emerging infections and disease emergence.

**DOI:** 10.3201/eid0502.990228

**Published:** 1999

**Authors:** M E Wilson


**Dan David,* Charles E. Rupprecht,† Jean Smith,† Itzhak Samina,* Shmuel Perl,* and Yehuda Stram***
*Kimron Veterinary Institute, Bet Dagan, Israel; and †Centers for Disease Control and Prevention, Atlanta, Georgia, USA

### References

1. Shimshony A. Veterinary public health in Israel. Revue Scientifique et Technique Office International Des Epizooties 1992;11:77-98.
2. Heaton PR, Johnstone P, McElhinnely M, Coweley R, O'Sullivan E, Whitby JE. Heminested PCR assay for detection of six genotypes of rabies and rabies related viruses. J Clin Microb 1997;35:2762-6.
3. Smith JS. Rabies virus. In: Murray PR, Baron EJ, Pfaller MA, Tenover FC, Yolken RH, editors. Manual of clinical microbiology. 6th ed. Washington: American Society for Microbiology; 1995. p. 907-1003.
4. Smith JS, Seidel HD, Warner CK. Epidemiology and historical relationships among 87 rabies virus isolates determined by limited sequence analysis. J Infec Dis 1992;166:296-307.

### Emerging Infections and Disease Emergence

**To the Editor:** Emerging infections have been defined as diseases whose incidence in humans has increased within the last 2 decades or threatens to increase in the near future (l). This definition, with minor variations, has continued to be used, although occasional debate erupts over whether one disease or another is truly emerging. Use of the term "emerging" has facilitated communication about the changed pattern of infectious diseases in recent years. While the study of infectious organisms and clinical training about emerging infections are manifestly necessary, they are not a sufficient foundation for understanding the process of disease emergence. I would propose, specifically, that we distinguish between emerging diseases—the study of specific infections that are changing—and the study of disease emergence.

Studies of emerging infections typically rely on disease, organismic, or syndromic approaches. Meetings on emerging infections typically cover newly recognized or characterized organisms or diseases and update information about the recognition, diagnosis, treatment, prevention, and control of these infections. This ongoing education is essential for practicing clinicians, who finished their formal training before AIDS, Lyme disease, ehrlichiosis, *Helicobacter pylori* infection, cryptosporidiosis, cyclosporiasis, and many other infections were described. These meetings also help clinicians learn how to fit new information into their existing knowledge base: What is the probability that a person with rash and fever has ehrlichiosis and that a person with fever and pulmonary infiltrates has hantavirus pulmonary syndrome?

By contrast, understanding the process of disease emergence involves studying the origins and ecology of emerging infections. Many disciplines relevant to disease emergence lie outside traditional infectious disease training and research and include evolutionary biology, demography, population dynamics, ecology, vector biology, climatology, epidemiology, genetics, veterinary medicine, and behavioral sciences (2). Infectious diseases of animals and plants have both a direct and indirect impact on human health. The study of infectious diseases in other species may provide important insights into understanding the process of disease emergence in humans. The study is also relevant to understanding the species-to-species spread of organisms.

Tools used to study and understand disease emergence include mathematical modeling, geographic information systems, remote sensing, molecular methods to study the genetic relatedness of organisms, and molecular phylogeny. Paleobiology, paleoecology, and studies that allow the reconstruction of past events may help inform future research and policy.

A major challenge is to reach people with relevant skills, knowledge, and experience and develop a coherent framework to advance the understanding of the process of disease emergence. No one institution, organization, or country can itself prevent or manage emerging infectious diseases.

In the study of emerging infections we focus on the organism, the patient, and the human population. The study of disease emergence must be at the systems level and must look at ecosystems, evolutionary biology, and populations of parasites and hosts, whatever their species. A primary goal should be to identify conditions or combinations or sequences of events that herald a changed pattern of infections so that preventive strategies can be used.

**Mary E. Wilson**
Mount Auburn Hospital, Cambridge, Massachusetts, USA; Harvard Medical School and Harvard School of Public Health, Boston, Massachusetts, USA

**References**

1. Lederberg J, Shope RE, Oaks SC Jr., editors. Emerging infections: microbial threats to health in the United States. Institute of Medicine. Washington: National Academy Press, 1992.
2. Wilson ME, Levins R, Spielman A, editors. Disease in evolution: Global changes and emergence of infectious diseases. Vol 4. New York: Ann N Y Acad Sciences; 1994.

### Malaria Control in South America

**To the Editor:** The article by Roberts et al. regarding DDT use and malaria in South America (1) correctly observes that health policy makers have shifted the emphasis of malaria control programs from vector control to case detection and treatment and that malaria control has been woefully underfunded in recent years. However, their conclusions that increased malaria is due to decreased spraying of homes with DDT and that DDT is still needed for malaria control do not withstand close scrutiny.

The authors did not mention several factors influencing malaria increase in recent decades, including growing antimalarial-drug resistance, the deterioration of public health systems responsible for malaria control, and large-scale migration to areas at high risk for malaria (e.g., almost all Brazilian malaria cases occur in the Amazon region) (2,3). Extradomiciliary malaria transmission, poor housing conditions, and human behavior in frontier areas such as the Amazon region limit the usefulness of insecticides. Thus, the deduction of causality between less house spraying with DDT and increased malaria incidence under those circumstances is questionable.

Roberts et al. have not actually linked increased malaria with eliminating DDT use but rather with eliminating house spraying altogether, without implementing effective alternatives. Malaria's recent decline in Brazil is due to a strategy that combines health education, aggressive case detection and treatment, and environmental management to eliminate *Anopheles* breeding sites (C. Catão Prates, unpub. data). A similar strategy has sharply reduced malaria incidence and deaths in Colombia (W. Rojas, unpub. data). In Mexico, use of two synthetic pyrethroid insecticides (deltamethrin and lambda cyhalothrin) for bed-net treatment and house spraying is controlling malaria at a much lower cost than the use of the alternative insecticides tried earlier and mentioned by Roberts et al. (4). Far from being pursued "without meaningful debate," the reduction and phaseout of DDT and other persistent organic pollutants is the subject of a 3-year United Nations–facilitated global negotiation process begun in June 1998.

Roberts et al. assert that DDT applied indoors does not move easily from the application site; however, a mass balance model indicates that 60% to 80% of the DDT ends up outdoors within 6 months (K. Feltmate, A model and assessment of the fate and exposure of DDT following indoor application [bachelor's thesis]. Ontario: Trent University; 1998). From there, DDT can be transported long distances in air, waterborne sediments, and biota, accumulating in humans and other nontarget species (5). Meanwhile, residents of sprayed houses accumulate high, persistent body levels of DDT through skin contact and food contaminated with DDT from air and dust (6).

Long considered a probable human carcinogen, DDT also is associated with reduced lactation, premature births, absorbed fetuses, and lower birth weights (7-9). In addition, recent animal research has raised the possibility that exposure of human fetuses or infants to DDT may cause permanent behavioral changes and impairment of body systems (10-12).

Synthetic pyrethroid insecticides used on bed nets or for house spraying against malaria-infected mosquitoes seem safer for human health than DDT because humans and other mammals possess the ability to hydrolyze the pyrethroids rapidly and excrete them from the body (13-14). Nevertheless, DDT and pyrethroids share known health risks, notably endocrine disruption, and the possible transgenerational consequences of chronic human exposure to pyrethroids have not yet been studied (10,15-16). Optimal protection of human health requires the development of integrated malaria control strategies that eliminate or reduce routine insecticide use by taking maximum advantage of environmental management, biological controls, and other nonchemical vector control measures (17).

